# Target splitting non-coplanar RapidArc radiation therapy for a diffuse sebaceous carcinoma of the scalp: a novel delivery technique

**DOI:** 10.1186/1748-717X-9-204

**Published:** 2014-09-16

**Authors:** Jiang Hu, WeiWei Xiao, ZhiChun He, DeHua Kang, ALong Chen, ZhenYu Qi

**Affiliations:** Sun Yat-sen University Cancer Center; State Key Laboratory of Oncology in South China; Collaborative Innovation Center of Cancer Medicine, Guangzhou, 510060 China; Department of Radiation Oncology, Sun Yat-Sen University Cancer Center, Guangzhou, 510060 China

**Keywords:** Total scalp irradiation, RapidArc, Target splitting, Non-coplanar, Dosimetry

## Abstract

**Background and purpose:**

To compare conventional lateral photon-electron, fixed-beam intensity modulated radiation therapy (IMRT), coplanar and non-coplanar RapidArc for the treatment of a diffuse sebaceous gland carcinoma of the scalp.

**Methods:**

Comprehensive dosimetry comparisons were performed among 3D-CRT, IMRT and various RapidArc plans. Target coverage, conformity index (CI), homogeneity index (HI) and doses to organs at risk (OAR) were calculated. Monitor unites (MUs) and delivery time of each treatment were also recorded to evaluate the execution efficiency. The influence of target splitting technique and non-coplanar planning on plan quality was discussed.

**Results:**

IMRT was superior to 3D-CRT concerning targets’ coverage at the sacrifice of larger irradiated brain volumes to low doses. CIs and HIs were better in coplanar RapidArc and non-coplanar RapidArc plans than 3D-CRT and IMRT. Best dose coverage and sparing of OARs were achieved in non-coplanar plans using target splitting technique. Treatment delivery time was longest in the IMRT plan and shortest in the coplanar RapidArc plan without target splitting. The 3%/3 mm gamma test pass rates were above 95% for all the plans.

**Conclusions:**

Target splitting technique and non-coplanar arcs are recommended for total scalp irradiation.

## Introduction

Sebaceous carcinoma of the scalp is rare, with very few cases reported in literature [1]. Radiotherapy has historically been proven an effective method for local treatment of sebaceous carcinoma, especially when surgery is not recommended [2]. However, delivering radiation for total scalp is technically challenging due to the concave shape and the proximity to critical structures. Traditional techniques such as stationary electron-beam fields may cause unacceptable hotspots in the field junctions [3, 4]. Utilizing lateral opposed photon fields matched with lateral electron fields and shifting the junction during the treatment are effective ways to improve dose uniformity at the junction [5], but target coverage is still unsatisfied.

Intensity modulated radiation therapy (IMRT) is potentially suitable for total scalp irradiation with the ability to produce a concave dose distribution. It has been demonstrated that fixed-beam IMRT can improve the target dose coverage and homogeneity compared to 3D-CRT [6]. Nevertheless, it decreases the brain volume irradiated to high doses at the cost of larger brain volumes irradiated to lower doses [6]. Recently, a rotational IMRT delivery technique, named RapidArc, was reported by Kelly et al. for total dural irradiation [7]. By using case-individualized collimator angle settings, they achieved a much better dose conformity with coplanar RapidArc than 9-field IMRT. This result may suggest that RapidArc can provide a more promising solution over fixed-beam IMRT for total scalp radiotherapy.

The purpose of this study is to explore the feasibility and efficiency of RapidArc in total scalp irradiation. The advantages of target splitting technique and non-coplanar planning were discussed with the aim to acquire an optimal radiotherapy modality for the treatment of a total scalp irradiation-like target volume.

## Methods

### CT simulation and target definition

From 2003, three patients diagnosed as low differentiated sebaceous gland carcinoma of the scalp were treated at our institution. These cases have typical spherical shell-shape tumor target with diffuse infiltration of skull and multiple nodules. Patients were immobilized with head-and-neck thermoplastic masks in a supine position. CT simulation was performed with 3 mm slice thickness and 3 mm slice spacing including the head and neck.

**Figure 1:**
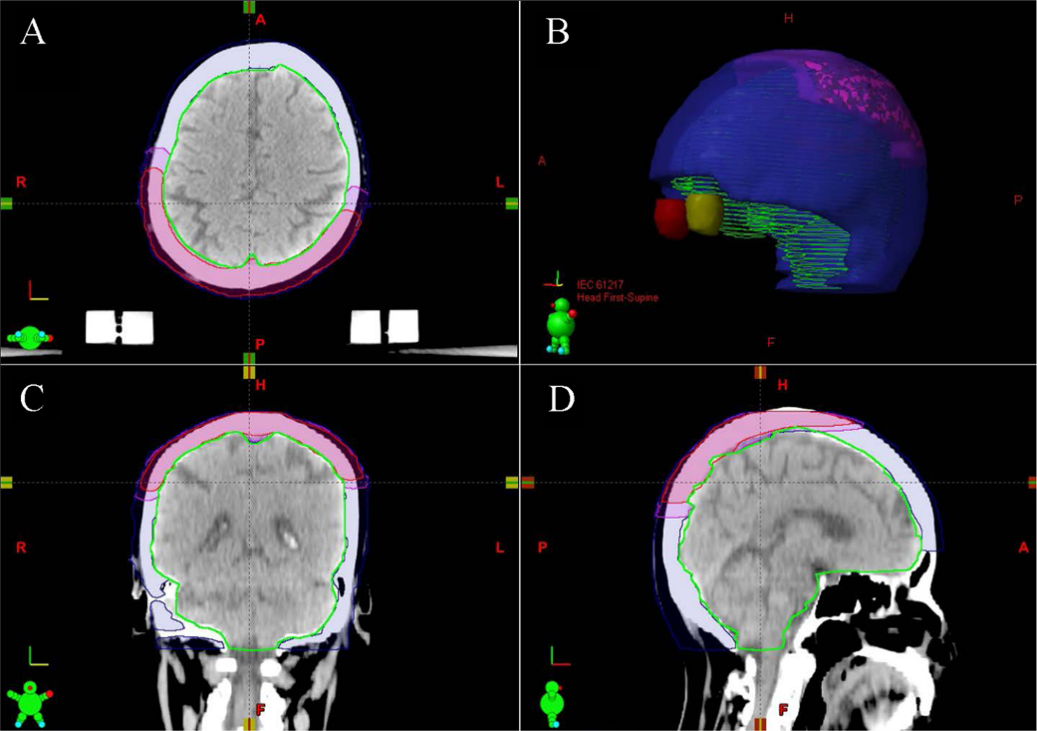
**Representative planning computed tomography (CT) slices. (A)** axial, **(C)** coronal, **(D)** sagittal and **(B)** three dimensional reconstructions. The PTV-G,PTV1 and PTV2 were rendered in blue, purple and red, respectively.

Tumor targets and key structures of interest were contoured on each CT slice in the Eclipse treatment planning system (Edition 10.0.1, Varian Medical Systems, Palo Alto, CA), following the guideline of the International Commission on Radiation Units and Measurements Reports 50 and 62 [8, 9]. Gross tumor volume (GTV) was delineated according to the physical examination findings, CT and MRI scan images, including cortex and medulla of parietal bone, and the maximum diameter is 61 mm × 53 mm × 31 mm. CTV1 was defined as the GTV plus a 10 mm margin (confined to scalp) and CTV2 was defined as the whole scalp. Additional isotropic 3-mm margins were added to GTV, CTV1 and CTV2, respectively, to create corresponding planning target volumes (i.e. PTV-G, PTV1 and PTV2) as shown in Figure 1. Normal tissues such as brain and optical structures were also contoured as organs at risk.

### Treatment planning

For comparison purpose, different treatment plans were generated by using Varian Eclipse treatment planning system, including 3D-CRT, 9-field IMRT and RapidArc plans. All the treatments were undertaken on a Varian Trilogy linear accelerator (Varian Medical Systems, Palo Alto, CA). Anisotropic Analytical Algorithm (AAA) model was used for dose calculation with a dose grid of 3 mm × 3 mm × 3 mm. Tissue heterogeneity corrections were also included.

#### 3D-CRT plan

The 3D-CRT plan was planned in three phases: 50 Gy in 25 fractions for the first phase (PTV2), 10 Gy in 5 fractions for the second phase (PTV1) and another 10 Gy in 5 fractions for the third phase (PTV-G). A treatment technique described by Akazawa [5] was applied in this study, which included two electron and two photon fields. The “skullcap” area was irradiated by parallel opposed 6 MV photon fields with a 1 cm thick wax bolus. The rest of the scalp was treated with two opposed 9 MeV electron beams matched to the upper photon fields. A 0.5 cm thick wax bolus was used in electron beams to build up skin dose and to protect the brain from electron dose.

#### 9-field IMRT plan

The IMRT plan was generated with nine equally spaced (0°, 40°, 80°, 120°, 160°, 200°, 240°, 280°, 320°) coplanar 6MV photon beams, which is similar to Wojcicka’s study [6]. A 1 cm thick wax bolus over the entire scalp was used to increase the superficial doses. Dose prescription was set to 70 Gy in 30 fractions to the PTV-G, 60 Gy in 30 fractions to the PTV1, and 50 Gy in 30 fractions to the PTV2. The optimization goals and constraints used for the PTVs and normal tissues were detailed in Table 1.

**Table 1:** **Optimization goals and constraints used for IMRT and RapidArc planning**

Structure	Prescription	Objective	Priority
PTV-G	70Gy/30F	V_95%_ ≥ 100%	600
		V_110%_ ≤ 10%	200
		D_max_ < 80Gy	600
PTV1	60Gy/30F	V_95%_ ≥ 100%	600
PTV2	50Gy/30F	V_95%_ ≥ 100%	600
Brain		D_max_ < 72Gy	500
		D_mean_: <30Gy and as low as possible	500
		V_28Gy_ < 30%	500
Len-Left		D_max_ < 10Gy	200
Len-Right		D_max_ < 10Gy	200
Eye-Left		D_max_ < 54Gy	100
Eye-Right		D_max_ < 54Gy	100
Optic never-L		D_max_ < 55Gy	100
Optic nerve-R		D_max_ < 55Gy	100
Chiasm		D_max_ < 55Gy	100

#### RapidArc plans

Three RapidArc plans were designed using the same dose prescription and optimization constraints as in the IMRT plan, including a standard RapidArc plan (sRapidArc), a split-target volume coplanar RapidArc plan (scRapidArc) and a split-target volume non-coplanar RapidArc plan (snRapidArc).

**Figure 2:**
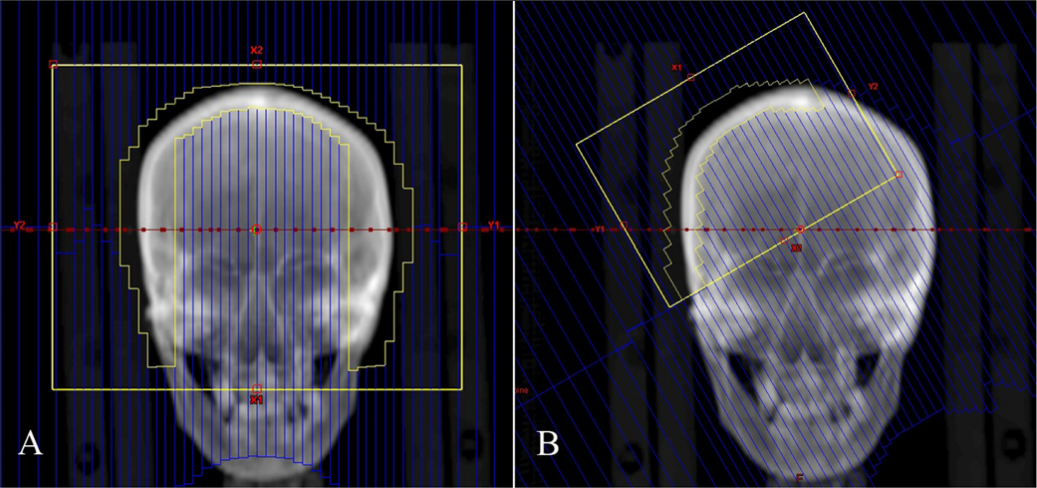
**Individualized collimator angle for total scalp irradiation. (A)** 90°as recommended by Kelly et al. [7]. **(B)** Splitting target from the middle vertical plane and 50°optimal collimator angle setting.

The sRapidArc plan included two coplanar full arcs with gantry rotating counterclockwise from 179° to 181° and clockwise from 181° to 179°. According to the recommendations of Kelly et al. [7], the collimator angles of two coplanar arcs were set as 90° with the aim of better shielding the normal brain tissues (Figure 2A).

As shown in Figure 2A, the spherical shell-shape target was quite difficult to achieve an optimal brain shielding with multileaf collimators (MLCs), even with the 90° collimator angle setting. Thus a split-volume treatment planning technique was developed by dividing the PTV2 into two parts from the middle vertical plane (Figure 2B). In the scRapidArc plan, the whole target was irradiated with two coplanar full arcs as in the sRapidArc plan. Another two coplanar partial arcs were specially introduced with optimal collimator angles (in this case, 50° to the left side and 330° to the right side), allowing the MLC best conform to the split-target so as to improve brain shielding.

In the snRapidArc plan, non-coplanar arcs with split-target technique were further evaluated for total scalp irradiation. The non-coplanar plan also included 4 arcs as in the scRapidArc plan, except for the gantry angles which were spatially distributed. The collimator angles were optimized in the three-dimensional directions to provide the best protection for normal brain tissues.

### Dosimetry analysis

The Conformity Index (CI) [10] was calculated using the following equation:


Where V_Tref_ was the volume of the target covered by the reference isodose, V_T_ was the target volume and V_ref_ was the volume of the reference isodose.

The Homogeneity Index (HI) [11] was defined as


Where D_x%_ was the absorbed dose received by x% of the target volume.

### Treatment delivery and dose verification

Patient specific dose verification was conducted with a 3D cylindrical diode array (ArcCHECK^TM^, Sun Nuclear, Melbourne, FL) for both IMRT and RapidArc plans. Hybrid phantom plans were created by re-computing the dose distribution with the QA phantom geometry using the same beam parameters of the patients’ plans. The discrepancies between the measured doses and TPS calculations were analyzed by using gamma-index method with a criterion of 3%/3 mm and a threshold dose of 10% of the maximum dose. MUs and delivery time were also recorded for each plan to evaluate the execution efficiency of different treatment techniques.

## Results

The dosimetric outcomes for five tested plans were listed in Table 2. Results showed that 3D-CRT failed to provide a satisfied dose distribution for PTVs in terms of either CIs or HIs due to the complex shape of the tumor target. V_95%_ was only 90.73%, 86.40% and 85.45% for PTV-G, PTV1 and PTV2, respectively. It also produced hotspots greater than 80 Gy (D_2%_ = 83.96Gy) in the abutting regions of photon and electron fields.

**Table 2:** **Dosimetric results for five treatment plans**

Structure	Dosimetry	3D-CRT	IMRT	sRapidArc	scRapidArc	snRapidArc
PTV-G	V_95%_ (%)	90.73	99.71	99.31	99.71	99.92
	V_110%_ (%)	14.18	12.86	16.74	0.07	0.97
	D_2%_ (Gy)	83.96	79.60	78.85	75.92	76.69
	CI	0.54	0.68	0.73	0.83	0.83
	HI	0.49	0.15	0.15	0.11	0.12
PTV1	V_95%_ (%)	86.40	100	99.99	99.50	99.72
	V_110%_ (%)	76.76	93.51	93.83	89.77	90.54
	D_2%_ (Gy)	82.39	79.23	78.73	75.80	76.53
	CI	0.43	0.45	0.47	0.54	0.54
	HI	1.13	0.22	0.21	0.21	0.21
PTV2	V_95%_(%)	85.45	99.54	99.70	99.80	99.50
	V_110%_ (%)	50.94	78.31	76.62	66.22	66.13
	D_2%_ (Gy)	78.81	77.76	77.83	75.12	75.75
	CI	0.59	0.74	0.77	0.83	0.83
	HI	1.42	0.47	0.47	0.45	0.47
Brain	D_1%_ (Gy)	69.66	68.70	66.07	62.36	62.03
	D_mean_ (Gy)	23.06	28.98	25.85	21.18	19.19
	V_5Gy_(%)	91.03	100	100	100	100
	V_10Gy_ (%)	60.98	100	79.34	58.12	50.14
	V_15Gy_ (%)	53.51	86.76	65.14	46.09	38.63
	V_20Gy_ (%)	47.28	52.46	50.99	37.24	31.36
	V_30Gy_ (%)	30.62	29.31	29.18	23.89	20.14
	V_40Gy_ (%)	21.01	16.92	16.65	12.64	10.21
	V_50Gy_ (%)	11.85	7.64	6.98	4.23	3.49
	V_60Gy_ (%)	4.39	3.33	2.24	1.14	1.12
	V_70Gy_ (%)	0.91	0.04	0	0	0
Eye-L	D_1%_ (Gy)	27.85	42.91	40.17	37.98	43.90
Eye-R	D_1%_ (Gy)	29.96	49.11	37.13	36.23	36.43
Len-L	D_1%_ (Gy)	10.52	16.02	10.84	10.23	10.28
Len-R	D_1%_ (Gy)	10.85	16.53	10.85	10.09	10.41
Never-L	D_1%_ (Gy)	7.10	36.82	30.16	31.06	35.38
Never-R	D_1%_ (Gy)	8.90	45.64	32.61	34.50	29.25
Chiasm	D_1%_ (Gy)	5.20	23.10	23.16	12.82	13.87

Both IMRT and RapidArc plans could offer a better dose coverage and homogeneity for target volumes compared with 3D-CRT. It was found that 95% of the prescription dose covered nearly 100% of PTVs in the IMRT and RapidArc plans. Also, D_2%_ was all within 80 Gy in these plans. Among them, improved dose conformity and homogeneity in the target volume were achieved with the split-target coplanar and non-coplanar RapidArc plans. V_110%_ accounted for less than 1% of PTV-G in the split-target coplanar and non-coplanar RapidArc plans, in contrast to 12.86% and 16.74% in IMRT and standard RapidArc plans.

It was seen from Table 2 that the IMRT plan was inferior to the 3D-CRT plan in sparing normal optical structures such as lens, eyes, optic nerves and chiasm. RapidArc plans could provide a comparable or even better protection for the lens than 3D-CRT, but failed in other optical structures. Nevertheless, our results demonstrated all the doses given to the optical structures were within clinically acceptable levels for both IMRT and RapidArc plans.

**Figure 3:**
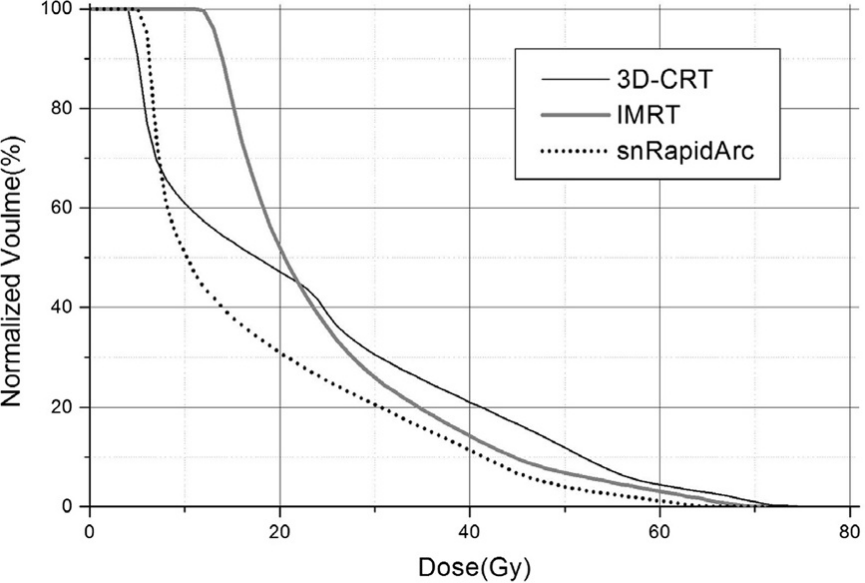
**An example of brain DVH curves (3D-CRT: black solid line, IMRT: gray solid line, snRapidArc: black dotted line).** The protective effectiveness for normal brain tissues was concluded to be snRapidArc > IMRT > 3D-CRT.

The normal brain tissue is a dose limiting organ for total scalp irradiation. As shown in Figure 3, the IMRT plan slightly decreased the high-dose irradiated volumes of the brain at the cost of larger volumes irradiated to lower doses compared to 3D-CRT. The mean dose to brain was 28.98 Gy in IMRT, higher than that in 3D-CRT (23.06 Gy).

The brain dose-volume parameters given in Table 2 clearly showed that the use of RapidArc, especially for the split-target coplanar and non-coplanar techniques, could further reduce the high doses delivered to the brain. The D_1%_ of brain was decreased from 69.66 Gy in 3D-CRT to about 66 Gy in the sRapidArc plan, and about 62Gy in both scRapidArc and snRapidArc plans. Beside this, it was found that the irradiated brain volumes from V_10Gy_ to V_70Gy_ in the snRapidArc plan were even smaller than those in 3D-CRT. The protective effectiveness for the brain tissue was thus concluded to be snRapidArc > scRapidArc > sRapidArc.

Monitor units and delivery time of each treatment plan were presented in Table 3. 3D-CRT and IMRT need the longest time (about 10 minutes). All three RapidArc treatments could be completed in less than half the time. Phantom measurements showed pass rates (3%/3 mm) above 95% for all the plans.

**Table 3:** **Dosimetric verification results for five treatment plans**

	3D-CRT	IMRT	sRapidArc	scRapidArc	snRapidArc
Monitor units	645	2832	756	1022	818
Delivery time (s)	620	636	156	264	352
Gamma criteria (%)	/	98.7	99.5	98.3	96.9

Also included were MUs and delivery time of each plan.

## Discussion

The goal of total scalp irradiation is to provide a uniform dose throughout the scalp while keeping the dose to the normal tissues as low as possible. But this therapeutic goal was not easily achieved owing to the concave shape of target and the close proximity of the target to OARs. In this work, a dosimetry comparison of 3D-CRT, fixed-beam IMRT, coplanar and non-coplanar RapidArc treatment techniques was undertaken for the patients diagnosed as low differentiated carcinoma of sebaceous gland in the scalp.

Lateral photon-electron technique was first reported in 1989 [5] and has been used in the treatment of scalp tumors for decades. Matching the photon and electron beams and shifting the match-line during the treatment course might yield acceptable dose coverage of targets and dose sparing of optical organs [12]. However, it could also cause hotspots of greater than 115% of the prescription dose in the fields’ junctions. This has been approved by our 3D-CRT plan, in which only suboptimal target dose coverage was obtained with hotspots in the abutting regions of photon and electron fields.

Substantial dosimetric advantages of IMRT over 3D-CRT have been proved in various cancers [13, 14]. More recently, dosimetric comparisons demonstrated IMRT could get consistent improvements in target coverage for scalp irradiation [6, 15]. We tried 5-field and 7-field coplanar and non-coplanar IMRT plans, but none could meet the dose optimization goal. Thus 9-field coplanar IMRT plan was used for comparison. It was found HIs were significantly decreased in the IMRT plan, compared to 3D-CRT. CIs were also increased from 0.54 to 0.68 for PTV-G, from 0.43 to 0.45 for PTV1 and from 0.59 to 0.74 for PTV2 in our study. These results agreed well with the previous findings reported by Wojcicka et al. [6]. However, as shown by Wojcicka et al. [6] and our study, IMRT provided little benefits to normal tissues in total scalp irradiation. Compared to 3D-CRT, the IMRT plan slightly decreased the high-dose irradiated volumes of the brain at the cost of larger volumes irradiated to lower doses. In addition, Wojcicka et al. [6] and our study both found that the 9-field IMRT plan even increased the D_1%_ delivered to optical structures than 3D-CRT.

Rotational therapies may suit to the delivery of scalp irradiation, with beamlets delivering tangentially to the scalp at all points. In Kelly’s study [7], case-individualized collimator angle of 90° was used to facilitate better shielding of the brain with MLCs. They found the case-individualized RapidArc plan compared favorably with the 9-field conventional IMRT plan. By using similar RapidArc designs, we obtained slightly better values of CI and HI in the sRapidArc plan. Compared to IMRT, the sRapidArc plan decreased the D_1%_ of all the optical structures, and foremost decreased the irradiated brain volumes from V_10Gy_ to V_70Gy_. However, the brain volumes irradiated to lower doses (from V_5Gy_to V_20Gy_) were still larger than 3D-CRT.

Target splitting has been reported to be effective in improving dose distribution, especially in large target volumes adjacent to normal tissues [16–19]. Sahgal et al. [16] found split-volume treatment planning techniques could significantly improve Cyberknife treatment plan quality for consecutive thoracic vertebral bodies’ irradiation than the standard full-volume technique. Similar results were reported by Seppälä et al. [17] in craniospinal irradiation. Wurstbauer et al. [18, 19] applied target splitting technique to deliver high dose to lung cancer and achieved a high level of locoregional tumor control and survival times. In this study, we further testified that the split-target technique was also suitable for total scalp irradiation. The scRapidArc and snRapidArc plans provided improved conformality and homogeneity for tumor target than sRapidArc and decreased the D_1%_ and D_mean_ of brain as well.

For typical spherical shell-shape target as shown in Figure 1, complete shielding of normal brain tissues couldn’t be achieved by using target splitting alone. Non-coplanar arcs may bring dosimetric advantages over static conformal beams especially for large and irregular targets, by allowing for more beam angel selection and more complete avoidance of normal tissue in three dimensional directions [20–23]. Here we applied the non-coplanar technique in the snRapidArc plan. Results showed CIs and HIs were largely the same as in scRapidArc, but D_1%_ and D_mean_ of the brain were further lower than scRapidArc. To our surprise, irradiated brain volumes from V_10Gy_ to V_70Gy_ were even smaller than 3D-CRT. In a previously published paper, Soisson et al. [24] also found dosimetric advantage of non-coplanar beam arrangements for treatment of skull-base tumors by reducing the size of low dose volumes of normal brain. Clinically, keeping the low dose volumes to normal brain to minimum might be of significance to limit possible cognitive impairment. As a result, the snRapidArc plan was selected as an optimal solution for total scalp irradiation. Phantom verification results show it can be executed accurately and efficiently.

## Conclusions

Considering all the dosimetric indices, target splitting and non-coplanar arcs are recommended for total scalp irradiation, which enable achieving more conformal and homogeneous targets’ dose coverage, lower brain dose, acceptable dose given to optimal structures and enough execution efficiency.
